# Predictors of One-Year Change in How Youth Perceive Their Weight

**DOI:** 10.1155/2020/7396948

**Published:** 2020-05-15

**Authors:** Karen A. Patte, Wei Qian, Scott T. Leatherdale

**Affiliations:** ^1^Department of Health Sciences, Brock University, 1812 Sir Isaac Brock Way, St. Catharines, Ontario L2N 3A1, Canada; ^2^School of Public Health and Health Systems, University of Waterloo, 200 University Avenue, Waterloo, Ontario N2L 3G1, Canada

## Abstract

Overall, perceptions of being at “about the right weight” appear advantageous for youth physical and mental health, regardless of BMI classification, whereas perceptions at either extreme (overweight or underweight) may negatively impact health behaviours and mental health. Instead of considering weight misperceptions as problematic, some researchers have proposed that underestimations of weight status may offer resiliency among individuals with overweight or obesity. Promoting “about right” WPs and preventing change to overweight or underweight perceptions may offer an effective public health strategy for supporting youth health over time. However, limited prospective evidence exists on factors that shape perceptions of weight status over time. The current study examined modifiable predictors of one-year change in weight perception among youths. We used 2-year linked data of 18,112 grade 9–12 students from Year 3 (Y_3_:2014–2015) and Year 4 (Y_4_:2015–2016) of the COMPASS study. Generalized Estimating Equation models tested screen use, physical activity, and bullying victimization as predictors of change from perceptions of “about the right weight” to “overweight” or “underweight” perceptions, adjusting for Y_3_ covariates (body mass index, ethnicity, and grade) and school cluster. Results support the value of team sports among females and resistance exercise among males as protective against changes to overweight or underweight perceptions over one year. Also, various forms of bullying victimization predicted overweight perceptions in males and females. Watching TV/movies or messaging/texting for over 2 hours/day was associated with overweight and underweight perceptions, respectively, in females only. Playing video/computer games for over 2 hours/day was associated with overweight perceptions in males and underweight perceptions in females. Findings support the potential of bullying prevention, limiting certain screen use, and supporting engagement in team sports for females and resistance exercise for males as strategies to maintain perceptions of being at “about the right weight.”

## 1. Introduction

Body image represents a multidimensional construct, encompassing how individuals view, feel, think, and act toward their physical appearance [1]. Weight perception (WP) refers to individuals' subjective appraisal of their body weight. In the research literature, misperceptions have been said to occur when there is a discrepancy between individuals' self-perceptions of their weight and their “objective” or measured weight status (as typically defined by body mass index (BMI) classification). WPs have long been studied in the eating disorders field and, more recently, emerged in the obesity literature. Several researchers became concerned that individuals with overweight or obesity may not perceive their weight as such and recommended correcting these perceptions to motivate weight loss [2–5]. Indeed, about one-third of youths report WPs that differ from their BMI classification [6–8]. However, consistent research indicates that such strategies are likely to have unintended and adverse consequences.

While overweight perceptions predict weight loss intentions [3, 7–11], several longitudinal studies have found perceptions of overweight to predict greater weight gain over time than “about right” perceptions [12–16]. Moreover, overweight perceptions are associated with less healthy diets, more sedentary behaviour, shorter sleep, less physical activity (PA), and an elevated risk of disordered weight-control behaviours (e.g., purging and fasting), in comparison to perceptions of being at “about the right weight” [4, 7–9, 15, 17–21]. Likewise, although they have received relatively limited research attention, perceptions of being underweight are shown to predict lower engagement in PA and less healthy diets (e.g., greater consumption of fast food, unhealthy snacks, sugar sweetened beverages, and energy drinks), than WPs of “about right.” [8, 21, 22].

Overweight and underweight perceptions also appear detrimental for youth mental health [23]. Prospective studies have found individuals who report being at “about the right weight” experience fewer depressive symptoms over time than their peers with overweight perceptions [24–26]. In fact, WPs partially account for many of the adverse psychosocial outcomes that have been associated with obesity (e.g., depression, suicidal ideation, and lower health-related quality of life) [3, 24, 27–32]. Similarly, underweight perceptions are associated with higher levels of depressive symptoms and social anxiety in males [23, 33, 34] and suicidality [27, 35] and lower health-related quality of life [29] in all youth.

Overall, perceptions of being at “about the right weight” appear advantageous for youth physical and mental health, regardless of BMI classification, whereas perceptions at either extreme (overweight or underweight) have negative effects on health behaviours and mental health. Instead of considering weight misperceptions as problematic, some researchers have proposed that underestimations of weight status may offer resiliency among individuals with overweight or obesity [26]. Promoting “about right” WPs and preventing change to overweight or underweight perceptions may offer an effective public health strategy for supporting youth health over time. However, the primary focus of WP research has been on correlates or outcomes of misperceptions, with limited prospective evidence on factors that shape perceptions of weight status. Existing literature largely consists of cross-sectional designs or differences by sociodemographic variables, but many associated variables may represent consequences of WPs rather than (or in addition to) predictors (e.g., differences in PA engagement) or are not modifiable to inform interventions (e.g., gender, age, ethnicity, and family/peer BMI). The purpose of the current study is to explore predictors of changes in WPs among youths. In particular, we examined whether bullying victimization, screen media use, and PA participation influenced the likelihood of youth changing from perceptions of being at “about the right weight” to reporting overweight or underweight perceptions over one year. We also examined whether these relationships differed by sex.

### 1.1. Screen Media Use

The *Tripartite Influence Model* posits exposure to three primary sources of influence, parents, peers, and media, contributing to the development of body image, mediated by appearance comparisons and internalization of societal appearance standards [36, 37]. Extensive body image research has examined the impact of media exposure, but the majority is exclusive to body dissatisfaction (i.e., the extent to which one experiences displeasure with his or her body) [38], rather than WPs and has been conducted in females only. The influence of screen media use likely varies by type and gender. Past body image literature largely centers around passive media viewing, such as print and television, where the concern was comparisons to celebrities and thin-idealized messages. In newer interactive forms, youth actively give and receive appearance-related feedback with peers. Extant research suggests internet use and social media have similar [39] or worse [40] effects on body satisfaction than traditional forms. Body comparisons to peers, and particularly with close friends, appear to have an equal to stronger influence on body ideals and satisfaction relative to that of celebrities [41–43]. On the other hand, social media may expose youth to the more varied body sizes of their peers, potentially having a normative effect on WPs. In support, experimental evidence shows that individuals view their bodies as smaller after exposure to normal or overweight images, relative to exposure to underweight images [44]. Female body image has generally been considered more responsive to social contexts and mass media [45–47], but this may reflect the focus on overweight concerns, without consideration of muscularity and underweight perceptions that are more common in males [6–8, 48].

### 1.2. Bullying Victimization

Screen use may influence WP not only through social comparisons but by exposure to cyber-bullying. Weight- or appearance-based teasing online or offline is a common experience during adolescence [49–51], reported by at least one-fifth of youth [49], and prospectively related to body dissatisfaction and monitoring in both boys and girls [52–54]. Compared to their peers, youth who have overweight and obesity are more likely to be targets of bullying and experience more frequent victimization [55–57]. Qualitative evidence suggests that underweight peers are also subject to teasing due to their underdeveloped physique, and boys report concerns about being targeted by stronger peers [58]. Some evidence suggests that links between weight status and victimization are attenuated when body satisfaction or perceived weight is accounted for [59–62]. Several cross-sectional studies demonstrate an association between bullying victimization and WPs of either overweight or underweight [61, 63–65]. Different forms of bullying engagement may lead youths to perceive their weight as overweight or underweight, with relationships potentially varied by gender, but no longitudinal studies have tested bullying as a predictor of WP changes.

### 1.3. PA Engagement

Body-related teasing and peer body surveillance often occur in the context of sports and other physical activities, where appearance tends to be more exposed [58, 66, 67]. Many cross-sectional studies have linked PA engagement to WPs [68, 69], which has typically been interpreted as WPs influencing PA engagement. Indeed, prospective evidence demonstrates underweight and overweight perceptions to predict lower PA than “about right” perceptions [8]; however, scant research has tested the reverse. A recent study found that adolescents who regularly practiced sports outside of school hours had more accurate body size perception than their peers not engaged in any extracurricular PA [70]. Concerns of adverse effects have typically centered around more weight-sensitive and aesthetically focused sports where thinness is considered advantageous, yet activities emphasizing muscularity also present risks for body image disturbance [71, 72]. In males, qualitative research suggests that sport and physical education settings provide important forums to compare their body to peers and discuss muscularity [58, 67, 73]. Adolescent boys report that an athletic, slim, strong, and muscular body aesthetic is necessary for sports participation, with those not conforming to this ideal subjected to teasing [67, 73]. Therefore, PA participation has the potential to be protective for WP or promote underweight or overweight perceptions, depending on context and gender.

## 2. Methods

### 2.1. Design

The COMPASS (Cannabis use, Obesity, Mental health, PA, Alcohol use, Smoking, Sedentary Behaviour) study is an ongoing (2012–2021) prospective study designed to collect hierarchical longitudinal data once annually from students in grades 9 through 12 and the secondary schools they attend [74]. School boards and schools were purposefully selected based on whether they permitted active-information passive-consent parental permission protocols [74], which are critical for collecting robust data among youth [75]. All grade 9 through 12 students attending participating schools were eligible to participate and could decline at any time. A full description of COMPASS and its methods are available in print [74] or online (http://www.compass.uwaterloo.ca). All procedures were approved by the University of Waterloo and Brock University Office of Research Ethics and appropriate school board committees.

### 2.2. Participants

The current study used linked student-level data from Year 3 (Y_3_: 2014–2015) and Year 4 (Y_4_: 2015–2016) of the study. In Y_3_, data were collected from 42,355 youth (79.3% participation rate) in 87 secondary schools in Ontario (*n* = 39,013 at 78 schools) and Alberta (*n* = 3342 at 9 schools). In Y_4_, data were collected from 40,436 students in 81 schools (9 Alberta schools; 72 Ontario schools (7 withdrew due to labour issues/illness, 1 new school)) [76]. Missing respondents resulted primarily from scheduled free/study periods or absenteeism during data collection.

To explore longitudinal changes among respondents, Y_3_ and Y_4_ student-level data were linked within schools (80 schools participated in both Y_3_ and Y_4_). The process of linking student data across waves is described in more detail by Qian and colleagues [77]. Due to the rolling sample design [74], it was not possible to link students who were in grade 12 at first participation and graduated that year or the grade 9 students that were newly admitted to participating schools in Y_4_. The other main reasons for nonlinkage included students transferring schools or dropping out, students not providing data for grade or sex (232), students on scheduled free/study periods or absent during data collection, or inaccurate data provided in the linkage measures. A total of 17,880 students were successfully linked for the two years of data collection. The final sample consisted of 17,475 youth, after removing students missing WP data for Y_3_ (237) and/or Y_4_ (184).

### 2.3. Data Collection Tool

Student-level data were collected using the COMPASS student questionnaire (Cq), a paper-based survey designed to collect student-reported data on multiple health behaviours, correlates, and demographic variables from full school samples during one classroom period. Cq items were based on the national standards or current national public health guidelines as described elsewhere [74]. The cover page contains measures to create a unique self-generated code for each respondent in a school to ensure the anonymity of participants, while still allowing COMPASS researchers to link each student's anonymous identifier data over multiple years [78].

### 2.4. Measures

#### 2.4.1. WP

Consistent with previous studies [8], WP was assessed by asking “how do you describe your weight?” Response options included the following: “very underweight,” “slightly underweight,” “about the right weight,” “slightly overweight,” and “very overweight.” Responses of “very underweight” and “slightly underweight” and of “very overweight” and “slightly overweight” were collapsed into “underweight” and “overweight,” respectively. Changes from perceptions of “about the right weight” to perceptions of “overweight” or to “underweight” were modelled.

#### 2.4.2. PA Measures

The PA measures have been previously validated [79]. To assess moderate-to-vigorous PA (MVPA), respondents were asked how many minutes of hard- and moderate-intensity PA they engaged in on each of the last 7 days. Consistent with the Canadian PA guidelines for youth [80], students were classified based on whether they had performed at least 60 minutes of daily MVPA on each of the last 7 days. Similarly, students were categorized based on whether they met the three times weekly recommendation for resistance exercise [81], by asking “on how many days in the last 7 days did you do exercises to strengthen or tone your muscles (e.g., push-ups, sit-ups, and weight training)?” Other PA items assessed whether respondents participated in competitive sports teams against other schools (e.g., varsity sports), league or team sports outside of school, and school-organized PA at noon, before, or after school (e.g., intramurals and noncompetitive clubs).

#### 2.4.3. Screen Use

Using previously validated measures [82], screen time was assessed by asking students the average time in hours and minutes per day that they spent: “watching/streaming TV shows or movies,” “playing video/computer games,” “talking on the phone,” “surfing the internet,” and “texting, messaging, emailing.” Responses were categorized based on whether they exceeded two hours per day on average for each type of screen use.

#### 2.4.4. Bullying Victimization

Bullying victimization was assessed by the following question: “in the last 30 days, in what ways were you bullied by other students? (mark all that apply).” Provided response options included the following: “I have not been bullied in the last 30 days,” “physical attacks (e.g., getting beaten up, pushed, or kicked),” “verbal attacks (e.g., getting teased, threatened, or having rumours spread about you),” “cyber-attacks (e.g., being sent mean text messages or having rumours spread about you on the internet),” and “had someone steal from you or damage your things.” Responses were dichotomized according to whether they reported having experienced each form of bullying in the last 30 days.

#### 2.4.5. Covariate and Stratifying Measures

Models were adjusted for student-reported race/ethnicity (white, nonwhite minority (black, Asian, indigenous [Métis, Inuit, First Nations], Hispanic/Latin American, others), grade (9–12), and BMI (kg/m^2^) category (recoded as underweight, normal weight, overweight/obesity, and missing). BMI classifications were determined based on student-reported height and weight [83] and the World Health Organization's [78] age- and sex-adjusted cutoff points. The weight status measure has been found to be reliable, valid, and valuable for use when objective methods are not feasible [84]. Missing BMI was included as category, due to the high amount of missing data (due to missing height, weight, age, or sex). Regression models were stratified by student-reported sex (male, female).

### 2.5. Analyses

All analyses were performed using the statistical package SAS 9.4. Descriptive statistics were calculated by sex. Generalized Estimating Equation (GEE) models were used, with independent working correlation for multinomial outcomes [85]. The GEE model is an extension of generalized linear models to correlated data, simply modelling the mean response and treating covariance as nuisance. It produces consistent estimates for regression parameters. Models tested Y_4_ measures of types of PA participation, bullying victimization, and screen use as predictors of changes to Y_4_ WPs of “overweight” or “underweight” (reference category: Y_4_ “about the right weight”), adjusting for Y_3_ covariates (grade, ethnicity, BMI classification), in youth with a Y_3_ WP of “about the right weight.” Models were stratified by sex and adjusted for Y_3_ covariates (grade, ethnicity, BMI classification). Schools were included in the models as clusters to take account of within-school correlation.

## 3. Results

### 3.1. Descriptive Statistics

Descriptive statistics for all variables in Y_3_ are presented in Table 1. The majority of the sample identified as white (72.6% females; 68.3% males) and 52.3% as indicated they were female. Females were more likely than males to report experiences of verbal bullying or cyber-bullying victimization in the last 30 days (16.9% vs. 11.9% and 7.5% vs. 2.2%, respectively), while males were more likely to report physical attacks (3.2% vs. 1.2%) or having their belongings stolen or damaged (3.1% vs. 2.3%) than females. More males than females met the MVPA (58.1% vs. 47.5%) and resistance exercise guidelines (57.4% vs. 50.3%) and played competitive (41.9% vs. 37.6%) and noncompetitive school sports (48.4% vs. 39.6%), while more females played sports outside of school than males (45.3% vs. 39.9%). A higher portion of males played video/computer games in excess of 2 hours a day (49.1% vs. 10.5%); females outnumbered males in exceeding 2 hours per day for all other forms of screen use.

Excluding students missing BMI data (18.5%), 19.8% of females and 32.4% of males had BMIs classified as overweight/obesity. Few youth had underweight BMI classifications (1.7% of females and 2.0% of males with BMI data). Over half of youth reported Y_3_ WPs of “about the right weight” (57.5% females; 55.9% males). Females were more likely to report “overweight” WPs than males (31.5% vs 22.2%), while males were about twice as likely to report “underweight” WPs than females (21.9% vs. 11.0%). Male WPs were about evenly divided between underweight and overweight (21.9% vs. 22.2%), while females were more likely to report overweight than underweight WPs (31.5% vs. 11.0%).

**Figure 1:**
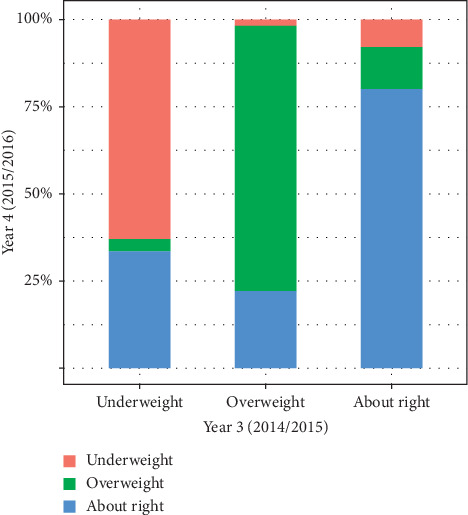
One-year change in weight perception (WP) among grade 9–12 students in the COMPASS study (percentage of WPs reported in Year 4 (2015/2016) by WPs reported in Year 3 (2014/2015)).

See Figure 1 for one-year changes in WP (percentage of WPs reported in Y4 by WPs reported in Y3).  Table 2 presents the frequency of Y_4_ WPs in students that reported Y_3_ WPs of “about the right weight.” Most females and males with Y_3_ WPs of “about the right weight” continued to perceive their weight as “about the right weight” (80.6% and 78.6%, respectively) in Y_4_. In students that changed from Y_3_ WPs of “about the right weight,” females were more likely to switch to “overweight” WPs than to “underweight” WPs (14.5% vs. 5.0%), while about the same proportion of males changed to WPs of “underweight” as to “overweight” in Y_4_ (11.4% vs. 10.1%).

### 3.2. GEE Models

In students with Y_3_ WPs of “about the right weight,” GEE models were used to test whether types of PA participation, bullying victimization, and screen use predicted WPs of “underweight” (see Table 3) and “overweight” (see Table 4) in Y_4_, with continued WPs of “about the right weight” as the reference category. Models controlled for grade, ethnicity, and BMI and adjusted for school clustering. Grade and ethnicity were not significant WP predictors in females or males. As expected, students with BMIs in the overweight/obesity category had higher odds of changing to overweight WPs in Y_4_ and were much less likely to change to underweight WPs. Likewise, underweight BMIs were strong predictors of changing from about right to underweight WPs in males and females. No females with underweight BMIs changed to WPs of overweight. Males with missing BMI data were more likely to report Y_4_ overweight WPs.

#### 3.2.1. Predictors of “Underweight” WPs in Y_4_ among Students with Y_3_ WPs of “about the Right Weight”

Table 3 presents the GEE model results for predictors of “underweight” WPs in Y_4_ among students with Y_3_ WPs of “about the right weight.” Females who participated in competitive school sports teams or on leagues/teams outside of school, and spent no more than 2 hours/day playing video/computer games or texting, messaging, or emailing, were less likely to change from WPs of “about the right weight” to WPs of “underweight” in Y_4_. In males, engaging in resistance exercise at least 3 times per week was associated with lower odds of “underweight” WPs at follow-up. Bullying was not associated with WP changes to “underweight” in females or males, and screen use did not predict “underweight” WPs in males.

#### 3.2.2. Predictors of “Overweight” WPs in Y_4_ among Students with Y_3_ WPs of “about the Right Weight”

Table 4 shows the GEE model results for predictors of “overweight” WPs in Y_4_ among students with Y_3_ WPs of “about the right weight.” Females who engaged in resistance exercise at least 3 times a week and watched television or movies for no more than 2 hours per day on average had slightly lower odds of changing from “about the right weight” WPs in Y_3_ to WPs of “overweight” in Y_4_, while experiences of verbal bullying or cyber-bullying victimization or having their belongings stolen or damaged increased the likelihood of “overweight” Y_4_ WPs. Males who met the MVPA and resistance exercise guidelines, participated in league/team sports outside of school, and played video/computer games for no more than 2 hours per day were less likely to change from WPs of “about the right weight” in Y_3_ to “overweight” WPs in Y_4_. Physical and verbal bullying victimizations were associated with increased odds of overweight WPs, while males who had experienced cyber-bullying were less likely to report overweight WPs.

## 4. Discussion

Evidence suggests that perceptions of weight are stronger correlates of health behaviours and mental health than objective weight status [3, 27–32]; therefore, understanding what factors contribute to the formation of WPs has important public health implications. However, while a wealth of literature exists on predictors of body satisfaction, negligible prospective research has explored on what contributes to perceptions of weight status. Using 2-year linked data from a large sample of secondary school students, this study examined whether bullying victimization, screen use, and PA predicted changes to overweight or underweight WPs among youth who previously reported describing their weight as “about the right weight.”

Consistent with previous studies [6–8], at baseline, more females described their weight as overweight than males and males were more likely than females to report underweight perceptions. About an equal proportion of males reported overweight as underweight WPs. Just over half of students reported “about right” WPs at baseline, leaving over 40% of youth at a greater risk for unfavourable health behaviour engagement and mental health, given evidence of adverse associations with both overweight and underweight perceptions [4, 8, 9, 23, 26, 29]. Approximately 80% of youth with baseline perceptions of “about the right weight” continued to perceive their weight this way one year later. In students who changed WPs, males were about evenly divided between changes to underweight and overweight perceptions, while females were more likely to report overweight than underweight perceptions at follow-up. Results align with female aesthetic body ideals of thinness and male body ideals of muscularity, which comprises both leanness and muscle size [48, 73, 81].

Student grade and ethnicity did not predict change in WP, while results for BMI were in the expected direction. That is, youths with “overweight/obesity” BMIs were more likely to change from “about the right weight” to “overweight” perceptions and less likely to change to underweight perceptions, relative to youth with “normal weight” BMIs. Youth with underweight BMIs were more likely to change from about right to underweight WPs than their peers with “normal weight” BMIs. Results for the missing BMI category were similar to overweight/obesity BMI in terms of higher odds of changing to overweight WPs than those with “normal weight” BMIs. Interestingly, overweight/obesity BMI appeared to have a stronger effect on WP change in males, while underweight BMI may have a stronger impact on WP change in females.

Participation on competitive school sports teams appears protective among females for maintaining WPs of “about the right weight” and against WP changes to either overweight or underweight. Females playing team/league sports outside of school were also less likely to shift to WPs of underweight. Apart from BMI, it is not known if females who played sports exhibited increased physical strength or altered body composition and if such changes played a role in the protective effect against WP changes. As models controlled for MVPA and BMI, results suggest that the protective effects result more from the context of competitive sports teams, perhaps through positive social interactions or self-identity, than simply due to the volume of PA or any possible weight change from participating. In a longitudinal study of body image development from adolescence to adulthood, females were more likely to report positive body image trajectories if they found a new social context in which they felt belonging and empowerment [86]. Sports team participation also encourages body functionality appreciation, as opposed to appearance appreciation, which has been shown to protect against exposure to thin-ideal images [84]. In an Australian study, adolescent girls on sports teams reported higher functional body satisfaction than their peers engaged in nonsports team PA or not physically active [87]. Similarly, in a study of girls aged 12 to 18, participation in stereotypically feminine sports was associated with feeling overweight, while participation in stereotypically masculine sports was not [88]. Apart from sports teams, PA is often motivated by weight loss and thinness goals in females, but no effect on WP change was found in the current study with MVPA and resistance exercise.

In males, engaging in resistance exercise at least three times per week, as per recommendations [80], was protective against changes to both underweight and overweight WP. Likewise, males were less likely to change to WPs of overweight if they met the MVPA recommendations of at least 60 minutes per day on average. Independent of PA volume, participation in league/team sports outside of school also had a protective effect against WPs of overweight. That is, more aerobic-oriented engagement predicted lower odds of overweight perceptions, while resistance exercise was protective for both overweight and underweight perceptions in males. As discussed, besides BMI, changes in physical strength or body composition were not assessed. Gaining muscle mass may contribute to the protective role of resistance exercise on WP. Males approach body image in a different way than females; in a longitudinal study of body image trajectories from adolescence to adulthood, males who developed positive body images had actively worked to improve their body shape and began to perceive themselves as resembling the ideal [86]. Results support the importance of various sports and PA engagement for the development of male WP [67, 73]. More active males and females are typically found to report higher body satisfaction [67, 87–90], with mixed results by context of PA. PA participation has the potential to also be protective for WP, with sex-specific effects by the type of engagement.

Various forms of bullying victimization predicted risks for overweight WPs in both females and males. Verbal bullying was the most common form of victimization and was associated with changes to overweight perceptions in all youths. About 17% and 11% of females and males reported experiencing verbal attacks within the last 30 days, respectively. In females, experiences of verbal bullying and cyber-bullying, and having their belongings stolen or damaged, increased the odds of overweight perceptions, while physical bullying was the least common form experienced and was not associated with WP changes. In males, verbal and physical bullying victimization were associated with greater risk of overweight perceptions. Weight- or appearance-related bullying is one of the most prevalent forms of victimization among youths. In a large Canadian study, 46.8% of girls reported someone saying something bad about their body shape, size, or appearance at least once in the last year, compared to 28.0% of boys [91]. Overall, while the current study is limited in knowing the target of the bullying, results fit with evidence of the prevalence of weight-based bullying, particularly verbal teasing, experienced by youth [55–57] and associations between bullying and WP independent of BMI classification [61, 63–65].

Interestingly, for reasons that are not immediately clear, cyber-bullying attacks predicted lower odds of changes to overweight perceptions in males but higher odds in females. Previous evidence indicates cyber-victimization is associated with body dissatisfaction and overweight preoccupation in male and female adolescents [92–95]. In a population-based study of Irish adolescents, males and females who had been cyber-bullied were almost twice as likely to consider themselves “too fat” as their peers who had not been victimized online [94]. Some studies report more body‐related concerns in girls who had experienced bullying compared to boys who were victims of bullying [53, 58, 63, 96]. Body shape, size, and weight appearance are primary targets of cyber-bullying [97, 98]. In a study of grade 6–9 students in British Columbia, Canada, about one-third of respondents claimed cyber-harassment because of their body [97]. As mentioned, the current study did not assess the target of the bullying. One study found that cyber-bullying focused on victim's appearance was associated with body-related concerns but cyber-bullying experiences in general were not [97]. Mixed results for cyber-bullying may reflect different gender-stereotypical body ideals. In a focus group of adolescents, girls reported receiving negative comments online about being “fat” and not fitting the thin ideals, while some boys reported experiencing cyber-bullying for not being muscular enough [96]. Results require replication in future studies that include measures regarding body- or appearance-targeted bullying.

We found that bullying victimization was not associated with changes to perceptions of underweight in males or females, consistent with research showing no association between being cyber-bullied and perceptions of being “too thin” among Irish adolescents; victimization was associated with concerns of being “too fat” [94]. The current study only assessed victimization and not perpetration. Based on previous studies [64, 65], the relationship with underweight or overweight perceptions may differ in youth who are victims from those who are both victims and perpetrators of bullying. In a cross-sectional study of British adolescents, neither bullying perpetration nor victimization were associated with objective weight, but youths reporting victimization had greater odds of overweight misperceptions and perpetration-victimization was linked to underweight misperceptions [65]. In contrast, a Slovak study found that adolescents dissatisfied with their weight due to feeling overweight were more likely to become passive or reactive victims, while self-reported thinness was associated with bullying victimization only in boys [64].

In females, watching no more than two hours of television/movies per day had a modest protective effect for changes to overweight perceptions. Results conflict with a recent longitudinal study, in which watching television was not significantly associated with body weight misclassification in an Australian adolescent cohort [99] but aligns with the vast body image literature on media [100, 101]. Female body image has generally been considered more responsive to mass media [45, 46]. Apart from portrayals of thin ideals, stigmatizing portrayals of obesity are pervasive across media types, including television, movies, print, and online [102, 103], with some evidence suggesting higher weight-stigmatizing content on youth-targeted media [103]. Exposure to weight- or shape-related teasing and teasing with overweight targets is inversely associated with adolescent girls' body satisfaction, regardless of viewers' weight status [104]. Interpretation of our results is limited by not knowing the content that adolescents were viewing. Alternatively, the effect found among females who reported spending less time watching television and movies may reflect their greater engagement in beneficial extracurricular or sporting activities.

As expected, talking on the phone demonstrated no association with WP in males or females, potentially given the lack of opportunity for visual social comparison. Surprisingly, no effect resulted for time spent surfing the internet or for texting, messaging, and/or emailing on risk for overweight perceptions, but the latter was associated with underweight perceptions in females. Controlling for cyber-bullying may have mitigated an effect of the internet or communication screen time on overweight perception in females. It is not immediately clear how respondents would classify the time spent on social-networking platforms on the measures used to assess screen use in the current study. As adolescents tend to use their mobile devices and social media for communication, rather than for health information [105], responses to time spent “texting, messaging, emailing” may reflect their use of these social networking platforms most closely, as opposed to time spent “surfing the internet.” How social media and screens are used is likely an important moderator, with social comparisons believed to be central to body image associations [106, 107]. Previous studies conducted in adolescent girls and women suggest that the impact of social media on body image outcomes is mostly detrimental, but is dependent on the context and feedback from peers [108–111]. Evidence generally supports a small-to-moderate effect of overall engagement in social-networking sites on body image, but appearance-specific functions such as photo sharing and viewing appear to have a much stronger impact [108–111]. The effect of messaging on females and not males fits with previous evidence showing a greater tendency for girls to engage in appearance-related conversations [112, 113]. Girls are also more likely to use social media to view others' photos and compare themselves with others, while males are more likely to use social media to find friends [114]. Also, in a qualitative study, girls emphasized surveillance of their peers' appearance via the photos they posted on social media and in school, while only in-school comparisons were reported by boys [58]. Both appearance-conversations and social comparisons with peers significantly predict body dissatisfaction [112, 115]. The increased risk of underweight perceptions in females, rather than overweight perceptions, with texting and messaging is surprising. While research has generally focused on the portrayal of thin-ideal messages, there has been a shift towards fitness-oriented depictions in the media, with the emergence of “fitspiration” and increasingly muscular portrayals [116], as well as greater representation of all body sizes. Both “thinspiration” and “fitspiration” are associated with lower body satisfaction [117]. More nuanced measures and analysis are required to determine the direction of screen media effects on WP. Our current measures are limited to time spent engaging in each type of screen use and do not inform the content or type of engagement.

Time spent playing video or computer games was the one form of screen use associated with WP changes in both female and male students. Females and males who played video/computer games for no more than 2 hours per day were less likely to change to perceptions of underweight or overweight, respectively. Some researchers suggest that the immersion in playing a character in video/computer games produces a more negative effect on body image than watching characters on television, which may be heightened in newer games with enhanced realism and that allow players to modify characters to closely resemble their actual or ideal appearance [118]. Several experimental studies have demonstrated an impact of playing video games with hyperideal videogame bodies on male and female body image [102]. Video games tend to reflect exaggerated aesthetic ideals; female characters are often hypersexualized, and male characters possess hypermuscular builds [119]. College-aged females and males reported lower body esteem after playing video games displaying muscular or thin characters, independent of BMI [118]. Men who played video games with exaggerated muscularity reported lower body satisfaction afterwards than a control group playing characters of average builds [120]. Interestingly, another study found that exposure to hyperideal video game bodies had a positive effect on women's body satisfaction and a negative effect on men's [119]. Overall, while concern has largely turned to the impact of social media on youth body image, results suggest television and movies remain important factors in shaping female WP, and video/computer gaming should be encompassed within efforts to promote positive body image.

Interpreting the current and existing research is limited by the construct of WP. Weight misperceptions are typically considered to occur when an individual's WP does not align with their BMI classification. However, when assessing WP, it is not known whether respondents answer by comparing their weight to an idealized body (i.e., where they want their weight to be), by assessing their weight relative to that of others (e.g., their peers, family, or people in the media), or by estimating where their weight falls on medical standards (e.g., BMI).

Responses of “about the right weight” may indicate body satisfaction rather than youth's evaluation of their weight relative to an external reference point (e.g., BMI). Some researchers question if weight misperception is truly lacking recognition of one's body weight and suggest that it may be closer to weight satisfaction (i.e., liking the weight one's body is at) [28]. In support, “about right” WPs are associated with lower body dissatisfaction [23], and body satisfaction demonstrates analogous associations with weight gain, health behaviours, and mental health, as found with “about right” WPs [121, 122].

The adverse effects associated with perceptions of overweight may result from internalized obesity stigma. Based on a laboratory-based study, individuals who perceive themselves as heavier are more susceptible to weight stigmatization messages [123]. This evidence, along with the literature demonstrating the adverse impacts of overweight perceptions, led some researchers to suggest that weight underestimations may offer resiliency among individuals at higher body weights [28]. That being said, in addition to targeting individual-level factors, upstream strategies are necessary to change the social norms that drive negative connotations of varying body weights. Adolescence represents an ideal age for interventions, as a critical period for the development of self-perceptions and body image [124, 125]. Substantial pubertal changes in height, weight, and body shape and composition occur alongside heightened social pressures and greater exposure to sociocultural ideals. Moreover, inaccurate perceptions of body weight are believed to begin in adolescence and continue into adulthood [99]. Increasing media literacy has been shown to reduce the impact of exposure on body satisfaction [126] and may also prove effective for adverse impacts on WP. In addition, schools are advised to implement bullying prevention strategies, avoid weight-targeted approaches, and promote PA engagement among all youth.

### 4.1. Strengths and Limitations

Key strengths of this study include the linked data and large sample of youth. Several limitations also require consideration. A total of 421 participants were removed due to missing WP responses, which may have been meaningful and linked to weight dissatisfaction. Self-reported weight data may not be missing at random [127]. For this reason, students with missing BMI data were retained, and missing BMI was included as a separate category. While sociodemographic variables were included as covariates, there remains the potential for residual confounding due to unmeasured confounders. Self-report measures are subject to recall and social desirability biases. As discussed, while BMI is controlled in the models, it does not measure body composition or distribution changes that may contribute to WP. Relatedly, BMI classifications are age- and sex-adjusted [78] but do not account for variations in lean and fat mass by factors such as ethnicity. Also, the small portion of students with BMIs classified as underweight prevents interpretation of these results. While the current study included ethnicity as a covariate, we were limited in exploring differences in relationships by sociocultural groups and intersectionality, given the largely Caucasian sample. Another limitation is the single-item measure used to assess WP, which limits the ability to detect the complex nature of this construct, and as mentioned, the reference point that youth use to respond is not entirely clear. Some misclassification on adherence to PA measures is probable. Furthermore, we do not know the content viewed when engaging in screen time, or the target of different forms of bullying. Also, only two waves of linked data were available at the time of this analysis. One-year change and the yearly interval between measurements may not be optimal to understand effects over time. Future analysis of the 9-year longitudinal COMPASS study will allow for examination of weight perception trajectories over a longer period of time and, in relation to mental health, weight loss intention, and appearance self concept, to help clarify mechanisms. It should be noted that majority of the reported effects persist, if *p* values were adjusted (e.g., to *p* < 0.01) to account for the multiple predictors. Finally, while the large sample supports generalizability, COMPASS was not designed to be representative.

## 5. Conclusions

Perceptions of being at “about the right weight” are shown to promote health behaviours and mental health among all youth, regardless of BMI. This study provides prospective evidence of modifiable predictors of WP changes among a large sample of youth. Future studies should explore the potential of bullying prevention, limiting certain forms of screen media use and supporting youth engagement in sports and resistance exercise, as strategies to maintain perceptions of being at “about the right weight” over time. However, upstream efforts are also needed to prevent and change the pervasive weight bias that likely drives the negative effects of overweight and underweight perceptions.

## Figures and Tables

**Table 1:** Year 3 (2014/2015) descriptive statistics among grade 9–12 students with 2-year linked data for Year 3 (2014/2015) and Year 4 (2015/2016) in the COMPASS study.

		Female (*n* = 9225)	Male (*n* = 8655)	Chi-square
		% (*n*)^b^	% (*n*)^b^	*p* value
Grade	9	35.7 (3295)	38.0 (3286)	<0.0001
	10	34.8 (3209)	34.5 (2989)	
	11	28.2 (2601)	25.6 (2214)	
	12	1.3 (120)	1.9 (166)	

Ethnicity	White	72.6 (6698)	68.3 (5911)	<0.0001
	Nonwhite	27.4 (2527)	31.7 (2744)	

BMI classification^a^	Underweight	1.3 (117)	1.6 (135)	<0.0001
	Normal weight	59.5 (5492)	51.9 (4490)	
	Overweight/obesity	15.0 (1383)	25.6 (2217)	
	Missing BMI	24.2 (2233)	20.9 (1813)	

WP	Underweight	11.0 (1006)	21.9 (1864)	<0.0001
	“About right”	57.5 (5237)	55.9 (4767)	
	Overweight	31.5 (2872)	22.2 (1897)	

PA				
School-organized PA (e.g., intramurals)	Yes	39.6 (3631)	48.4 (4135)	<0.0001
	No	60.4 (5535)	51.6 (4415)	
School league/team sports (e.g., varsity)	Yes	37.6 (3445)	41.9 (3588)	<0.0001
	No	62.4 (5710)	58.1 (4974)	
League/team sports outside of school	Yes	47.5 (4338)	58.1 (4975)	<0.0001
	No	52.5 (4797)	41.9 (3587)	
Met MVPA guidelines^c^	Yes	47.5 (4338)	58.1 (4975)	<0.0001
	No	52.5 (4797)	41.9 (3587)	
Met resistance exercise guidelines^c^	Yes	50.3 (4601)	57.4 (4900)	<0.0001
	No	49.7 (4552)	42.6 (3644)	

Screen time				
TV/movies	≤2 hours/day	43.6 (4012)	47.6 (4112)	<0.0001
	>2 hours/day	56.4 (5200)	52.4 (4526)	
Video/computer games	≤2 hours/day	89.5 (8248)	50.9 (4398)	<0.0001
	>2 hours/day	10.5 (964)	49.1 (4240)	
Surfing the Internet	≤2 hours/day	48.1 (4432)	61.7 (5327)	<0.0001
	>2 hours/day	51.9 (4780)	38.3 (3311)	
Talking on the telephone	≤2 hours/day	89.1 (8204)	94.0 (8117)	<0.0001
	>2 hours/day	10.9 (1008)	6.0 (521)	
Texting/messaging/emailing	≤2 hours/day	48.1 (4429)	67.6 (5842)	<0.0001
	>2 hours/day	51.9 (4780)	32.4 (2796)	

Bullying victimization in the last 30 days				
Physical	Yes	1.2 (107)	3.2 (273)	<0.0001
	No	98.8 (9118)	96.8 (8382)	
Verbal	Yes	16.9 (1557)	11.9 (1031)	<0.0001
	No	83.1 (7668)	88.1 (7624)	
Cyber	Yes	7.5 (692)	2.2 (191)	<0.0001
	No	92.5 (8533)	97.8 (8464)	
Stolen/damaged belongings	Yes	2.3 (214)	3.1 (265)	<0.0001
	No	97.7 (9011)	96.9 (8390)	

^a^BMI classification based on self-reported height and weight and age- and sex-adjusted cutoffs. ^b^Numbers may not add to total due to rounding and/or missing data. ^c^Based on PA guidelines for youths doing at least 60 min/day of MVPA and strengthening exercises at least 3 days/week.

**Table 2:** Weight perceptions (WPs) in Year 4 (2015/2016) of the COMPASS study among grade 9–12 students with Year 3 (2014/2015) WPs of “about the right weight.”

Year 4 (2015/2016) WP	Females (*n* = 5185) % (*n*)	Males (*n* = 4722) % (*n*)
Underweight	5.0 (257)	11.4 (536)
About the right weight	80.6 (4177)	78.6 (3711)
Overweight	14.5 (751)	10.1 (475)

**Table 3:** GEE model results for predictors of youth perceiving their weight as “underweight” in Year 4 (2015/2016) of the COMPASS study among grade 9–12 students with Year 3 (2014/2015) WPs of “about the right weight.”

	Females (*N* = 5062)			Males (*N* = 4557)		
	OR	95% CI	*p* value	OR	95% CI	*p* value
WP: underweight (ref.: about the right weight)						

Covariates						
Grade (9–12 continuous)	1.05	0.90, 1.22	0.5329	0.91	0.81, 1.02	0.0921
Ethnicity (white vs. nonwhite)	0.92	0.69, 1.24	0.6037	1.07	0.86, 1.34	0.5423
BMI category (ref.: “normal weight” BMI)^a^						
Underweight BMI	**6.30**	**3.06, 12.95**	<0.**0001**	**3.16**	**1.71, 5.85**	**0.0002**
Overweight/obesity BMI	**0.06**	**0.01, 0.40**	**0.0035**	**0.22**	**0.14, 0.32**	<0.**0001**
Missing BMI	0.69	0.47, 1.00	0.0507	0.85	0.65, 1.12	0.2595

Physical activity (yes vs. no)						
Met MVPA guidelines^b^	1.07	0.83, 1.38	0.6171	0.92	0.79, 1.08	0.3052
Met resistance exercise guidelines^b^	1.03	0.75, 1.40	0.8758	**0.77**	**0.64, 0.92**	**0.0040**
Varsity sports	**0.66**	**0.48, 0.91**	**0.0109**	0.98	0.75, 1.27	0.8604
School-organized PA/intramurals	0.99	0.67, 1.47	0.9510	1.07	0.86, 1.33	0.5530
League/team sports outside of school	**0.72**	**0.54, 0.95**	**0.0222**	0.98	0.81, 1.20	0.8576

Bullying victimization (yes, in the last 30 days vs. no)						
Physical attacks	1.38	0.52, 3.65	0.5130	1.26	0.68, 2.34	0.4617
Verbal attacks	1.48	0.99, 2.23	0.0567	1.32	0.95, 1.84	0.1020
Cyber-attacks	0.78	0.42, 1.42	0.4118	1.05	0.60, 1.84	0.8710
Stolen/damaged belongings	1.50	0.60, 3.76	0.3878	1.42	0.80, 2.53	0.2307

Screen time (≤2 hours/day vs. >2 hours/day)						
Watching TV/movies	1.06	0.80, 1.40	0.6817	0.92	0.79, 1.07	0.2749
Playing video/computer games	**0.62**	**0.45, 0.87**	**0.0053**	0.85	0.67, 1.08	0.1800
Talking on the phone	0.74	0.53, 1.04	0.0803	0.89	0.67, 1.19	0.4473
Surfing the Internet	0.88	0.65, 1.18	0.3852	0.94	0.78, 1.13	0.4808
Texting/messaging/emailing	**0.73**	**0.56, 0.95**	**0.0204**	0.88	0.72, 1.06	0.1826

The values in bold represent statistically significant results (p<0.05). All models were adjusted for school clustering. WP = weight perception; PA = physical activity; MVPA = moderate-to-vigorous physical activity; GEE = General Estimating Equations; BMI = body mass index; BMI classification based on self-reported height and weight and age- and sex-adjusted cutoffs. ^b^Based on PA guidelines for youth doing at least 60 minutes of MVPA per day on average and strengthening exercises at least 3 days a week.

**Table 4:** GEE model results for predictors of youth perceiving their weight as “overweight” in Year 4 (2015/2016) of the COMPASS study among grade 9–12 students with Year 3 (2014/2015) WPs of “about the right weight.”

	Females (*N* = 5062)			Males (*N* = 4557)		
	OR	95% CI	*p* value	OR	95% CI	*p* value
WP: overweight (ref.: about the right weight)						

Covariates						
Grade (9–12 continuous)	0.88	0.76, 1.01	0.0696	1.15	0.94, 1.42	0.1828
Ethnicity (white vs. nonwhite minority)	0.98	0.75, 1.29	0.9027	0.88	0.65, 1.18	0.3919
BMI category (ref.: “normal weight” BMI)^a^						
Underweight BMI^b^						
Overweight/obesity BMI	**3.49**	**2.41, 5.05**	<0.**0001**	**6.07**	**4.42, 8.34**	<0.**0001**
Missing BMI	**1.48**	**1.11, 1.98**	**0.0083**	**2.17**	**1.51, 3.12**	<0.**0001**

Physical activity (yes vs. no)						
Met MVPA guidelines^c^	0.89	0.74, 1.06	0.1791	**0.74**	**0.59, 0.91**	**0.0057**
Met resistance exercise guidelines^c^	0.85	0.70, 1.03	0.1050	**0.62**	**0.50, 0.78**	<0.**0001**
Varsity sports	**0.81**	**0.66, 0.99**	**0.0397**	0.89	0.70, 1.15	0.3785
School-organized PA/intramurals	0.96	0.78, 1.17	0.6817	1.18	0.90, 1.55	0.2199
League/team sports outside of school	0.91	0.74, 1.12	0.3783	**0.70**	**0.58, 0.85**	**0.0003**

Bullying victimization (yes, in the last 30 days vs. no)						
Physical attacks	1.80	0.87, 3.75	0.1157	**3.11**	**1.75, 5.55**	**0.0001**
Verbal attacks	**1.38**	**1.08, 1.76**	**.0089**	**1.74**	**1.30, 2.31**	**0.0002**
Cyber-attacks	**1.37**	**1.02, 1.84**	**.0351**	**0.29**	**0.11, 0.76**	**0.0117**
Stolen/damaged belongings	**1.85**	**1.00, 3.42**	**.0482**	1.01	0.50, 2.06	0.9675

Screen time (≤2 hours/day vs. >2 hours/day)						
Watching TV/movies	**0.85**	**0.73, 1.00**	**0.0443**	0.88	0.70, 1.10	0.2520
Playing video/computer games	0.87	0.68, 1.12	0.2796	**0.72**	**0.59, 0.89**	**0.0024**
Talking on the phone	0.97	0.77, 1.23	0.8163	1.09	0.81, 1.48	0.5657
Surfing the internet	0.89	0.75, 1.06	0.1911	0.86	0.69, 1.06	0.1496
Texting/messaging/emailing	0.95	0.80, 1.13	0.5640	1.03	0.86, 1.24	0.7150

The values in bold represent statistically significant results (p<0.05). All models adjusted for school clustering. WP = weight perception; GEE = General Estimating Equations; PA = physical activity; MVPA = moderate-to-vigorous physical activity; BMI = body mass index. ^a^BMI classification based on self-reported height and weight and age- and sex-adjusted cutoffs. ^b^No students with underweight BMIs changed from ”about right“ to ”overweight“ WPs. ^c^Based on PA guidelines for youth doing at least 60 minutes of MVPA per day on average and strengthening exercises at least 3 days a week.

## Data Availability

COMPASS study data are available upon request to the corresponding author or through an online form (https://uwaterloo.ca/compass-system/information-researchers/data-usage-application).
